# Physical activity, screen viewing time, and overweight/obesity among Chinese children and adolescents: an update from the 2017 physical activity and fitness in China—the youth study

**DOI:** 10.1186/s12889-019-6515-9

**Published:** 2019-02-15

**Authors:** Zheng Zhu, Yan Tang, Jie Zhuang, Yang Liu, Xueping Wu, Yujun Cai, Lijuan Wang, Zhen-Bo Cao, Peijie Chen

**Affiliations:** 1 Shanghai Research Center for Physical Fitness and Health of Children and Adolescents, Shanghai University of Sport, 399 Chang Hai Road, Shanghai, 200438 China; 2 School of Kinesiology, Shanghai University of Sport, 399 Chang Hai Road, Shanghai, 200438 China; 3 School of Physical Education and Sport Training, Shanghai University of Sport, 399 Chang Hai Road, Shanghai, 200438 China

**Keywords:** Body mass index, Sedentary behavior, Surveillance, Unhealthy lifestyle

## Abstract

**Background:**

With increases in inactive lifestyles and mounting pressure for academic excellence in Chinese younger populations, lack of physical activity and increased prevalence of obesity have become a major public health concern in China. The purpose of this study is to provide updated estimates on the prevalence of meeting moderate-vigorous physical activity (MVPA) and screen viewing time guidelines, and overweight and obesity among Chinese school-aged children and adolescents, with a secondary aim examining variations in prevalence by sex, grade groupings, and residential location.

**Methods:**

The study sample came from the 2017 Physical Activity and Fitness in China—The Youth Study, a cross-sectional and national survey of 131,859 students (aged 7 to 19 years) from 986 public schools in China. Measures of MVPA, screen viewing time, and age- and sex-specific overweight and obese body mass index were used to calculate national prevalence estimates of Chinese school-aged children and adolescents.

**Results:**

In 2017, 34.1% (95% confidence interval [CI], 34.09–34.11%) of children and adolescents met MVPA guidelines and 65.4% (95% CI, 65.39–65.41%) adhered to screen viewing time guidelines. The prevalence of overweight and obesity was 15.1% (95% CI, 15.09–15.11%) and 10.7% (95% CI, 10.69–10.71%), respectively. Prevalence estimates differed by sex (boys, girls), grade grouping (primary schools, junior middle schools, junior high schools), and residential location (rural, urban).

**Conclusions:**

There remains a low prevalence of meeting MVPA guidelines and high prevalence of overweight and obesity in Chinese school-aged children and adolescents. Future efforts should focus on monitoring the trend of these behavioral and health risk factors to inform school policies and programs aimed at increasing physical activity and reducing and preventing obesity in younger populations in China.

**Electronic supplementary material:**

The online version of this article (10.1186/s12889-019-6515-9) contains supplementary material, which is available to authorized users.

## Background

There is substantial evidence showing the health benefits of physical activity for children and adolescents [1, 2]. Despite this, the overall physical activity level in younger populations remains significantly low globally, and the prevalence of physical inactivity continues to be a major public health problem both in developed and developing countries. Studies indicate that children and adolescent populations in most countries show a low prevalence of overall physical activity levels [3–7], a high prevalence of sedentary behavior levels [5, 8], and a rising level of prevalence of obesity [9, 10]. Globally, approximately 80% of adolescents (ages 13–15 years old) are not meeting recommended guidelines [11], that is, they are spending less than 60 min of their daily time participating in moderate-vigorous physical activity (MVPA), including vigorous activities on at least 3 days per week.

China follows the global trend in developing major health risk factors in its younger populations [12–15]. A large-population school-based survey, the Physical Activity and Fitness in China—The Youth Study (PAFCTYS) [16], showed that, in 2016, with an average 45 min daily MVPA reported, approximately 70% of school-aged children and adolescents did not meet the recommended guidelines of 60 min of MVPA daily [17], 94% underachieved in meeting the Chinese national physical fitness “excellent” standards [18], 37% failed to adhere to the daily screen viewing time recommendations of 2 h or less, and 12% were classified as obese based on body mass index percentile scores [19]. Some differences in these health outcomes were noted between boys and girls, adolescents (in junior middle and junior high schools) versus young children (in primary schools), and children and adolescents living in rural communities versus those living in urban communities.

In order to promote healthy lifestyles and physical fitness, in 2016, China initiated its *Healthy China 2030* blueprint [20], which includes the health goals of having school-aged children achieve at least 1 h of physical activity daily and having more than 25% of them achieve an “excellent” rating in fitness. However, there are significant challenges and barriers to making progress toward meeting these public health goals. These include (a) a steady prevalence of physical inactivity in young populations; (b) a continued upward trend in obesity [13, 14]; (c) an overemphasis on academic excellence [21, 22], which has become an increasingly unhealthy cultural norm; and (d) a lack of cultural-specific and age-appropriate physical activity and/or intervention guidelines for school-aged children and adolescents. These issues have made the public health surveillance of China’s physical activity and other health outcomes a high priority, and addressing these issues is a necessary step toward establishing baseline data to inform policies and health promotion strategies.

Therefore, our primary aim in this study was to provide an update on the population prevalence estimates of MVPA, screen viewing time, and overweight/obesity among Chinese school-aged children and adolescents using data collected from the 2017 PAFCTYS. A secondary aim of the study was to examine variation in prevalence by sex, grade grouping, and residential location of the students.

## Methods

### Study design

The study sample was drawn from the 2017 PAFCTYS completed in December 2017. The overall design and methodologies employed in PAFCTYS have been provided elsewhere [17]. Briefly, PAFCTYS is a nationally representative annual surveillance study designed to assess the physical and health status of the Chinese school-aged children and adolescent population across three grade groupings in primary education: primary, junior middle, and junior high schools, as defined by the Chinese school education system [23]. The sampling scheme ensured a balanced representation of the country’s geographic areas, economic development, and rural-urban diversity. The student sampling took place in classes selected randomly from each grade in the selected schools within the previously determined sampling areas. This study was approved by the Ethics Review Committee of Shanghai University of Sport in 2017. As the study presented no more than minimal risk to participants, given the size and cultural background of the study populations, the Committee waived the need for written consent and approved the use of verbal consent.

### Study participants

Participants in the 2017 surveillance were 131,859 children from 490 primary schools, 251 junior middle schools, and 245 junior high schools recruited from a total of 31 provinces (*n* = 21), direct-controlled municipalities (*n* = 4), and regions (*n* = 6). The province of Tibet was excluded from the 2017 survey. The student ages ranged from 7 to 19 years old.

### Procedures

The verbal consent protocol approved by the Ethics Review Committee required researchers to obtain permission to conduct the study from teachers and principals of each participating school by detailing the purposes, potential risks, and benefits of the study participation prior to data collection. The same protocol was used with parents/guardians and their consent for their children’s participation was sought. Finally, all student participants were similarly educated about the research project prior to participation and their assent sought. Each verbal consent/assent was logged, by a research assistant, into a subject file with a numeric identification code and, subsequently, entered into a computer database accessed exclusively by authorized project staff.

Data collection took place during school term time between October and December 2017. Participating children were given detailed instructions on how to fill out the survey and were provided ample time for questions. Following a standardized survey administration protocol [17], trained research assistants conducted a survey of physical activity and assessment of body weight and height during prearranged regular school hours. The stadiometers and scales used in obtaining the weight and height data were calibrated before use. Students completed the survey either online (68%) or on a paper version (32%) in a classroom setting.

### Measures

#### Physical activity

Measures of MVPA were obtained from the adapted measure of the International Physical Activity Questionnaire (IPAQ)—Short Form [24]. Because of the limitations associated with all self-report questionnaires [25], there is a lack of documented evidence on the psychometric properties of our physical activity measure. However, we chose to use it for MVPA measurements for reasons of consistency with our past work on physical activity prevalence [17, 26, 27] and because previous empirical studies have shown that there are associations between children’s MVPA and family support and engagement and physical activity characteristics in school and community settings [28, 29].

The adapted scale used two items to specifically record the frequency and duration of engaging in moderate (e.g., biking, brisk walking) and vigorous (running, heavy weight-lifting) intensity levels of activities that lasted for 10 min or more in the past 7 days. The total minutes spent over the 7 days on moderate and vigorous exercise were summed and divided by 7 to create the mean minutes each child spent each day in MVPA [17]. Consistent with the WHO guidelines for children and youth [30] and the 2016 PAFCYTS report [17], the prevalence of adherence to meeting physical activity recommendations for 2017 was determined based on whether participants reported 60 min or more of MVPA per day.

#### Screen viewing time

Participants were asked to report the amount of screen viewing time (in hours) they spent, both on weekdays to *none*, *about a half hour*, *1 h*, *2 h*, or *3 h or more*. Consistent with previous studies [19, 27, 31], scores from these 3 daily items were converted into hours spent daily and were categorized into meeting (i.e., reported 2 h or less per day) or not meeting (more than 2 h daily) screen viewing time guidelines.

#### Body mass index (BMI)

Children’s body weight (kg) and height (cm) were measured in bare feet, using a portable instrument (i.e., GMCS-IV; Jianmin, Beijing, China). From these measurements, BMI for each participant was calculated as body weight (kg) divided by height (m) squared (kg/m^2^). BMI age- and sex-adjusted referenced points for overweight and obese were determined using the national reference norm for overweight and obesity established for the Chinese school-aged male and female children aged 7 through 18 [32, 33] (Additional file 1: Table S1). Once BMI was normalized by age and sex, participants were categorized into overweight and obese classes, with overweight being defined as having a BMI ≥ the referent age- and sex-specific 85th percentile but less than the 95th percentile and obesity as having a BMI ≥ the referent age- and sex-specific 95th percentile [15, 19].

#### Demographic measures

Information on participants’ age, sex, grade level, and residential location (rural, urban) was ascertained during the survey.

### Data analysis

Participants were included in the prevalence estimation if they had complete data for valid outcome data. Those who provided invalid data or had missing values on measures of physical activity, screen viewing time, or height or weight measurement were excluded from the analyses. Descriptive statistics were calculated for all study outcome variables. For prevalence, estimates were calculated as the percentage of children and adolescents who met the guidelines of 60 min of daily MVPA, adhered to screen viewing time guidelines, and were overweight and obese.

In a pre-specified protocol, analyses on prevalence were performed on the whole sample and then stratified by sex (boys vs girls), grade grouping (primary, junior middle, junior high schools) and residential location (rural vs urban). Differences in sex, grade grouping, and residential location were tested using binominal logistic regression models with and without adjustments for demographic covariates (i.e., sex, age, ethnicity grades, rural vs urban residence). Prevalence estimates and odds ratios (ORs) from the logistic regression models with their corresponding 95% confidence intervals (CIs) were reported. Tests were considered statistically significant at an overall *α* level of 0.05. No Bonferroni correction was made based on the a priori analysis plan.

Prevalence estimates of the 2017 PAFCTYS sample were weighted according to the Chinese school-aged population of children in the current school education system (i.e., primary, junior middle, and junior high). All statistical analyses were conducted using SPSS software version 15.0 (SPSS Inc., Chicago, IL, USA). Prevalence estimates were reported as percentages with 95% CIs.

## Results

In total, 131,859 school-aged children participated the 2017 study. The overall unweighted participation response rate for children and adolescents was 99%, calculated as the number of children and adolescents who participated (in both surveys and physical assessment on body weight and height) divided by the total number of targeted school children (*N* = 131,992). Of the total sample, 5402 (4%) of the children who provided no data on the study outcome measures were removed. An additional 21,034 students (16%) who had extreme (erroneous) responses on physical activity measures (i.e., responses exceeding 120 min of daily MVPA) and 177 (0.1%) with missing data on body weight and height were also removed from the current study.

After removing participants with missing data information and invalid data (*n* = 26,613), the final study included an effective sample size of 105,246 children and adolescents, which is similar to the 2016 PAFCTYS effective sample size [17]. The sample has a mean age of 13.33 years old (SD = 2.63; range: 7–19 years of age), 52% (54,689) were girls, and had a high Han ethnic representation (86%). Tables 1 and 2 report unweighted sample sizes and weighted percentages of the study population and descriptive statistics for the study population, respectively.

**Table 1:** Unweighted sample sizes and weighted percentages for Chinese children and adolescents aged 7 to 19 years: PAFCTYS 2017

	All grade grouping	Primary	Junior middle	Junior high
Both sexes	105,246 (100%)	33,342 (100%)	35,233 (100%)	36,671 (100%)
Girls	54,689 (52.1%)	17,357 (51.1%)	18,474 (52.4%)	18,858 (51.4%)
Boys	50,557 (47.9%)	15,985 (47.9%)	16,759 (47.6%)	17,813 (48.6%)
Residential location				
Urban	57,190 (53.7%)	17,744 (53.2%)	18,377 (52.2%)	21,069 (57.5%)
Rural	48,056 (46.3%)	15,598 (46.8%)	16,856 (47.8%)	15,602 (42.5%)

PAFCTYS denotes Physical Activity and Fitness in China—The Youth Study. Percentage is weighted using PAFCTYS sampling weights to be representative of the Chinese grade school population in 2017 (unweighted sample size = 105,246; weighted sample size = 93,099,259)

**Table 2:** Descriptive characteristics of Chinese school-aged children and adolescents aged 7 to 19 years: PAFCTYS 2017

Characteristic	Unweighted	Weighted
Age, mean, SD (y)	13.33 (2.63)	12.71 (2.49)
Ethnicity, percentage		
Han Others	85.9 14.1	85.7 14.3
Height, mean (SD) (cm)	157.12 (12.43)	155.13 (12.44)
Weight, mean (SD) (kg)	50.15 (13.93)	48.21 (13.75)
BMI	19.97 (3.63)	19.68 (3.63)
MVPA, mean (SD) (min.)	45 (31.51)	47.38 (31.67)
Screen viewing time, mean SD (min.)	113.16 (93.52)	114.72 (93.47)

PAFCTYS denotes Physical Activity and Fitness in China—The Youth Study. Percentage is weighed using PAFCTYS sampling weights to be representative of the Chinese grade school population in 2017 (unweighted sample size = 105,246; weighted sample size = 93,099,259)

*SD* standard deviation

*BMI* body mass index, calculated as body weight (kg) divided by height (m) squared (kg/m^2^)

*MVPA* daily moderate-vigorous physical activity

Detailed prevalence estimates of MVPA, screen viewing time, overweight, and obesity among Chinese children and adolescents in 2017 for the whole sample and by sex, grade grouping, and residential location are shown in Table 3. Overall, more than one-third (34.1%) of Chinese school children and adolescents reported meeting the recommended daily 60 min MVPA and more than two-thirds of the population (65.4%) reported adherence to the 2 h or less of screen viewing time recommended in the guidelines. The prevalence estimates indicate that about 15.1% of the Chinese children and adolescents were overweight and 10.7% were obese.

**Table 3:** Weighted prevalence of meeting of MVPA and screen viewing time guidelines, and overweight and obesity among Chinese school-aged students by sex, grade grouping, and residential location, PAFCTYS 2017

	meeting MVPA guidelines (95% CI)	meeting screen viewing time guidelines (95% CI)	Overweight (95% CI)	Obesity (95% CI)
Both sexes	34.10 (34.09–34.11)	65.40 (65.39–65.41)	15.10 (15.09–15.11)	10.70 (10.69–10.71)
Sex				
Girls Boys	31.90 (31.89–31.91) 36.50 (36.49–36.51)	67.70 (67.69–68.71) 62.80 (62.75–63.85)	12.40 (12.39–12.41) 18.0 (17.99–18.01)	8.30 (8.29–8.31) 13.20 (13.19–13.21)
Grade grouping				
Primary Junior middle Junior high	38.50 (38.48–38.52) 35.30 (34.28–35.32) 24.20 (23.18–24.22)	67.70 (67.68–67.72) 60.50 (60.48–60.52) 69.70 (69.68–69.72)	13.40 (13.39–13.41) 13.31 (13.09–13.11) 21.60 (21.58–21.62)	11.50 (11.49–11.51) 7.90 (7.89–7.91) 13.90 (13.88–13.92)
Residential location				
Rural Urban	34.10 (34.09–34.11) 34.10 (34.09–34.11)	64.00 (63.99–64.01) 66.60 (66.59–66.61)	14.10 (14.09–14.11)15.90 (15.89–15.91)	9.99 (9.89–9.91) 11.30 (11.29–11.31)

Prevalence is weighed using PAFCTYS sampling weights to be representative of the Chinese grade school population in 2017

*CI* confidence interval

There were statistically significant differences in the prevalence estimates by sex, grade grouping, and residential location (Table 4). Logistic regression model results indicated that boys had significantly higher odds of meeting the recommended 60 min daily of MVPA compared with their counterpart girls (OR = 1.23, *P* < 0.001). Relative to children in primary schools, adolescents in junior middle and junior high schools were less likely to meet the MVPA recommendations (OR = 0.87, *P* < 0.001; OR = 0.51, P < 0.001, respectively). There was no difference between children and adolescents living in either urban or rural communities.

**Table 4:** Differences in the prevalence of meeting of moderate and vigorous physical activity and screen viewing time guidelines, overweight, and obese by sex, grade grouping, and residential location, PAFCTYS 2017

	Meeting MVPA guidelines	Meeting screen viewing time guidelines	Overweight	Obese
	OR (95% CI)	OR (95% CI)	OR (95% CI)	OR (95% CI)
Sex				
Girls Boys	Referent 1.231 (1.230–1.232)	Referent 0.805 (0.804–0.806)	Referent 1.539 (1.537–1.541)	Referent 1.690 (1.688–1.692)
Grade grouping				
Primary Junior middle Junior high	Referent 0.871 (0.870–0.872) 0.511 (0.510–0.512)	Referent 0.728 (0.727–0.729) 1.095 (1.094–1.096)	Referent 0.979 (0.977–0.980) 1.787 (1.784–1.789)	Referent 0.660 (0.659–0.661) 1.240 (1.238–1.242)
Residential location				
	Referent 0.997 (0.996–0.997)	Referent 1.117 (1.116–1.118)	Referent 1.154 (1.152–1.155)	Referent 1.167 (1.165–1.168)
Rural Urban				

On the prevalence of screen viewing time, boys were less likely than their counterpart girls to meet screen viewing time guidelines (OR = 0.81, *P* < 0.001). Relative to children in primary schools, adolescents in junior middle schools were less likely to meet the guidelines (OR = 0.73, P < 0.001), and, in contrast, adolescents in junior high schools were more likely to meet the guidelines (OR = 1.1, P < 0.001). Children and adolescents living in urban settings were more likely to meet screen viewing time guidelines than those living in rural communities (OR = 1.12, P < 0.001).

On the outcomes of overweight and obesity, boys had significantly higher odds of being overweight compared with their counterpart girls (OR = 1.54, P < 0.001). Adolescents in junior high schools were significantly more likely than children in primary schools to be overweight (OR = 1.79, P < 0.001), but adolescents in junior middle schools showed a lower likelihood of being overweight relative to children in primary schools (OR = 0.98, *P* < 0.001). Children and adolescents living in urban communities were more likely to be overweight compared to those living in rural communities (OR = 1.15, P < 0.001). A similar pattern of findings was observed from the logistic regression model results on obesity, with higher odds of being obese among boys (compared with girls), junior high school adolescents (compared with children in primary schools), and children and adolescents living in urban communities (compared with those living in rural communities), and lower odds of being obese among adolescents in junior middle schools (relative to children in primary schools). Estimates from the logistic regression models with adjustments for covariates are presented in Additional file 1: Table S2. Although the adjustments on covariate made no significant changes on most outcomes, it was observed that, on the prevalence of overweight and obesity, adolescents in junior high schools were significantly less likely than children in primary schools to be overweight (OR = 0.53, P < 0.001) and obese (OR = 0.34, P < 0.001) relative to children in primary schools.

## Discussion

Using a nationally representative survey of Chinese school-aged children and adolescents, we reported updated prevalence estimates of meeting daily MVPA and screen viewing time guidelines, and overweight and obesity in 2017. These data provide a consistent and relatively flat trend in these behavioral and health outcomes among Chinese school-aged children and adolescents. Compared to the prevalence estimates in 2016 [17], estimates from 2017 showed a slight upward move in the proportion of students meeting MVPA guidelines (34.1% versus 29.9% in 2016) and meeting screen viewing time guidelines (65.4% versus 63.2% in 2016). On the population’s prevalence of overweight and obesity, a slightly upward trend (1%) in overweight and downward trend (1.2%) in obesity were observed in the 2017 estimates (15.1% for overweight, 10.7% for obesity) compared to the prevalence estimates [19] in 2016 (14% for overweight, 11.9% for obesity).

Stratified analyses indicated that prevalence estimates differed by age, grade grouping, and residential location. Boys had a significantly higher prevalence of meeting MVPA guidelines but a lower prevalence of meeting screen viewing time guidelines, and a higher prevalence of overweight and obesity than girls. Among the three grade groupings, adolescents in both junior middle and junior high schools tended to be less likely to meet MVPA guidelines compared to younger children in primary schools, and those in junior high schools were more likely to meet screen viewing time guidelines whereas those in junior middle schools were less likely to meet them. While adolescents in junior middle schools were less likely to be overweight and obese, those in high schools were more likely to be obese. Across urban and rural community living settings, children and adolescents living in the urban communities had a higher prevalence of overweight/obesity compared to those living in rural communities.

Overall, the updated prevalence estimates from 2017 show a continued and worrisome level of reduced physical activity, a higher level of sedentary behavior spent on screen viewing, and a steady level of high prevalence of overweight and obesity among school-aged children and adolescents. Prior research indicates a consistently low level of physical activity [17, 34] and an increased level of overweight and obesity [14, 15] among Chinese younger populations. Lack of sufficient physical activity and excess screen viewing time can all lead to negative health outcomes, such as increased risk for developing overweight or obesity [35, 36] and cardiometabolic risk [37]. There has been some compelling evidence that suggests that some of these childhood health risk factors, if not modified early on, may track into adulthood [38].

Cumulative findings from the existing literature and the findings from this study call for urgent actions for policy changes at the school, community, and clinical levels, as well as strategic public investments aimed at promoting physical activity [8, 16, 39]. While comprehensive intervention strategies that include a component of physical activity show reduction in overweight and obesity among Chinese school-aged children [40], there are currently no effective physical activity enhancement strategies to counter-balance the continued and chronic trend in physical inactivity and high prevalence of overweight and obesity among Chinese school-aged children and adolescents. There is also a clear public health need to address the continued disparities in behavioral health outcomes observed between urban and rural communities.

The present study has some notable weaknesses. First, data were collected in a single season (i.e., October/December). Therefore, the surveillance data may only reflect physical behaviors at a specific time of year and may not be generalizable to the entire calendar year. Second, the cross-sectional design is a limitation, and therefore the direction of causality cannot be inferred. Prospective longitudinal studies of surveillance aimed at describing the prevalence, trajectory, and determinants of physical activity, screen viewing time, and other health outcomes are necessary to understand the trend and patterns of change over time. Third, we acknowledged the limitations with our use of self-reported physical activity, which is known to result in overestimating or underestimating true energy expenditure or rates of sedentary behavior [25]. Likewise, the use of IPAQ for young children has not been rigorously evaluated for reliability and validity. These limitations together call for caution in interpreting our findings and for the need to conduct studies that evaluate the measurement properties of IPAQ in assessing children’s physical activity. Last but not least, to reduce the amount of erroneous data, approximately 20% of the data was removed due to the out-of-range values on the measures of physical activity. This resulted in a loss in the data and may have possibly affected the generalizability of the findings. However, the removal of these outlying values from our analyses was necessary as they tend to introduce bias and obscure point estimates [17].

Findings from this study and others conducted in China, as well as studies from the international community [5, 14, 15, 17, 19], have provided important epidemiological evidence that can be used to guide the development of policies and programs aimed at promoting physical activity among school-aged children both in school (within schools) and community (outside school) settings. Currently, there are no specific guidelines or initiatives that specifically target this population of school children and youth in China. Given the surveillance data from the current study and other Chinese data accumulated over the last decade, development of strategic prevention efforts are needed to increase physical activity time, reduce screen viewing time, curb unhealthy weight, and improve fitness levels among Chinese school children and adolescents. In addition, interventions targeting school children must be planned and developed quickly with public support and investments from local government, the private sector, and community stakeholders. Such intervention efforts should consider the important roles that policies [41], government strategies [5, 42], school practices [29], community and built environments [43, 44], and parents of children [28] play in facilitating participation in physical activity, suggesting that a multilevel preventive strategy is needed to step up the effort to help children and adolescents live active and healthy lives.

## Conclusion

In conclusion, the prevalence of MVPA, screen viewing time, and overweight and obesity among Chinese school-aged children and adolescents in 2017 is comparable to the estimates reported in 2016. These updated estimates fully justify the need for high prioritization of the promotion of physical activity as an important public health mission. The continued use of PAFCTYS to surveil and monitor these behavioral health outcomes is also needed in order to guide policies and programs aimed at increasing physical activity and reducing obesity among Chinese school-aged children and adolescents.

## Additional file

Additional file 1: **Table S1.** Age- and sex-specific body mass index reference norm cut-off values for overweight and obesity for Chinese boys and girls in different ages. **Table S2.** Differences in the prevalence in meeting moderate and vigorous physical activity (MVPA) and screen viewing time guidelines, overweight, and obese by sex, grade grouping, and residential location. The data information in this additional file serves as supplementary materials to the study. (DOCX 19 kb)

## Abbreviations

BMI: Body mass index
CI: Confidence interval
IPAQ: International physical activity questionnaire
MVPA: Moderate-vigorous physical activity
PAFCTYS: Physical activity and fitness in China-The Youth Study
