# Supplementary material for: Physical activity, screen viewing time, and overweight/obesity among Chinese children and adolescents: an update from the 2017 physical activity and fitness in China—the youth study
Source: BMC Public Health. 2019 Feb 15;19:197. doi: 10.1186/s12889-019-6515-9 (PMC6376726; doi:10.1186/s12889-019-6515-9)
Supplement: Supplementary file 1 — Table S1. Age- and sex-specific body mass index reference norm cut-off values for overweight and obesity for Chinese boys and girls in different ages. Table S2. Differences in the prevalence in meeting moderate and vigorous physical activity (MVPA) and screen viewing time guidelines, overweight, and obese by sex, grade grouping, and residential location. The data information in this additional file serves as supplementary materials to the study. (DOCX 19 kb) [file 12889_2019_6515_MOESM1_ESM.docx]

**Supplement**

Supplement to the paper by Zheng Zhu, Yan Tang, Jie Zhuang, Yang Liu, Xueping Wu, Yujun Cai, Lijuan Wang, Zhen-Bo Cao, Peijie Chen. Physical activity, screen viewing time, and overweight/obesity among Chinese children and adolescents: an update from the 2017 Physical Activity and Fitness in China—The Youth Study

**Content List (in the order of appearance in the paper)**

Supplement Table S1. Age- and sex-specific body mass index (BMI) reference norm^1^ cut-off values for overweight and obesity for Chinese boys and girls in different ages

Supplement Table S2. Differences in the prevalence of meeting of moderate and vigorous physical activity (MVPA) and screen viewing time guidelines, overweight, and obese by sex, grade grouping, and residential location

Supplement Table S1. Age- and sex-specific body mass index (BMI) reference norm^1^ cut-off values for overweight and obesity for Chinese boys and girls in different ages

| Age (in years) | Boys - overweight | Boys - obese | Girls - overweight | Girls - obese |
| --- | --- | --- | --- | --- |
| 7 | 17.4 | 19.2 | 17.2 | 18.9 |
| 8 | 18.1 | 20.3 | 18.1 | 19.9 |
| 9 | 18.9 | 21.4 | 19.0 | 21.0 |
| 10 | 19.6 | 22.5 | 20.0 | 22.1 |
| 11 | 20.3 | 23.6 | 21.1 | 23.3 |
| 12 | 21.0 | 24.7 | 21.9 | 24.5 |
| 13 | 21.9 | 25.7 | 22.6 | 25.6 |
| 14 | 22.6 | 26.4 | 23.0 | 26.3 |
| 15 | 23.1 | 26.9 | 23.4 | 26.9 |
| 16 | 23.5 | 27.4 | 23.7 | 27.4 |
| 17 | 23.8 | 27.8 | 23.8 | 27.7 |
| 18 | 24.0 | 28.0 | 24.0 | 28.0 |

^1^Established on the basis of percentiles and Z-scores reference data and percentile curves of BMI for age for boys and girls aged 7-18 years old. These age- and sex-specific values are used to categorize children and adolescents into overweight and obese classes. Overweight is defined as a BMI equal to or greater than the referent age- and sex-specific 85th percentile values but less than the 95th percentile, and obesity is defined as a BMI equal to or greater than the referent age- and sex-specific 95th percentile [1,2].

References:

1. Ji CY. BMI screening standards of overweight and obesity in Chinese students. Zhongguo Xue Xiao Wei Sheng. 2004;25:125-8. Chinese.

2. Group of China Obesity Task Force. Body mass index reference norm for screening overweight and obesity in Chinese children and adolescents. Zhonghua Liu Xing Bing Xue Za Zhi. 2004;25(2):97-102. Chinese.

Supplement Table S2. Differences in the prevalence of meeting of moderate and vigorous physical activity (MVPA) and screen viewing time guidelines, overweight, and obese by sex, grade grouping, and residential location

|  | Meeting MVPA guidelines | Meeting screen viewing time guidelines | Overweight | Obese |
| --- | --- | --- | --- | --- |
|  | OR (95% CI) | OR (95% CI) | OR (95% CI) | OR (95% CI) |
| Sex^1^  Girls  Boys | Referent  1.232 (1.231-1.233) | Referent  0.812 (0.811-0.813) | Referent  1.517 (1.515-1.519) | Referent  1.662 (1.660-1.664) |
| Grade grouping^2^  Primary  Junior middle  Junior high | Referent  0.787 (0.786-0.788)  0.416 (0.414-0.417) | Referent  1.062 (1.060-1.064)  2.330 (2.323-2.337) | Referent  0.532 (0.531-0.533)  0.528 (0.526-0.530) | Referent  0.342 (0.342-0.343)  0.335 (0.333-0.336) |
| Residential location^3^  Rural  Urban | Referent  1.011 (1.01-1.011) | Referent  1.106 (1.105-1.107) | Referent  1.158 (1.156-1.159) | Referent  1.183 (1.182-1.185) |

^1^Controlling for age, ethnicity, grades, and residential location.

^2^Controlling for age, sex, ethnicity, and residential location.

^3^Controlling for age, sex, ethnicity, and grades.
